# Blended Therapies and Mobile Phones for Improving the Health of Female Victims of Gender Violence

**DOI:** 10.3390/healthcare10030445

**Published:** 2022-02-26

**Authors:** Yolanda García, Carlos Ferrás

**Affiliations:** 1 Faculty of Education and Social Work, University of Vigo, 32004 Ourense, Spain; yolanda.garcia.vazquez@usc.es; 2 Mujeres Vulnerables Lab, Institute of Studies and Development of Galicia (IDEGA), University of Santiago de Compostela, 15782 Santiago de Compostela, Spain

**Keywords:** blended therapy, gender-based violence, mobile phones, mood, psychosocial health, medication adherence

## Abstract

We carried out a case study with a group of female victims of gender violence (*n* = 39) with the objective of evaluating a blended psychotherapeutic intervention. The results show that blended therapies with mobile text messages combined with face-to-face group therapies significantly improved the health of these women. Mood states and the symptoms of depression were measured with a PHQ-9 personal health questionnaire and evolved positively. In the group of women (*n* = 39) the scores improved from an initial 13.5 (SD = 7.2, range 3–34) to 6.0 at the end of the period of messages (SD = 5.2, range 0–18), which was a significant difference (t (39) = 2.02; *p* = 0.000). Most of the women stated that the messages had helped them improve their mood (91.6%) and their general health (83.3%), which made them feel more connected to their social environment (80.6%). We observed that adherence to medication for each woman improved. With mixed therapies and mobile phones, social service professionals can incorporate technology into daily practices and offer personalized attention and daily counseling to victims of gender-based violence.

## 1. Introduction

According to Baker et al. [1], Peláez et al. [2], and Bullock and Colvin [3], social services professionals need to adopt a practice-based approach to integrate information technologies and mobile communications into their professional activity. Such technologies are becoming increasingly pervasive, and their use in social services needs to be discussed and understood in depth [4,5,6,7], as well as their ethical implications and how negative attitudes towards technology in social work can be challenged [8].

In psychology-based approaches, experimental studies were conducted on online mental health therapies using text messages to the mobile phones of patients suffering from depression, anxiety, alcoholism, addictions, etc., combining such online therapies with traditional face-to-face care [9,10,11,12,13,14,15,16,17]. There is a general agreement that text messages reinforce the tasks and the advice received from face-to-face psychotherapies, promoting self-awareness and patient progress.

Mobile phone-based information and communication technologies are the most accessible forms of mediated communication in history, and texting is currently one of the most widely used forms of communication [18]. We must bear in mind that face-to-face communication promotes a strong therapeutic bond and an atmosphere of trust between social services professionals and users, but these can be complemented by online communication solutions that facilitate individual care [1]. Granholm [4] believes that there is space to develop the blended social work method, which combines face-to-face and online care. However, Cwikel and Friedman [19] and Bullock and Colvin [3] observed that social services professionals show little interest in integrating communication technologies and e-therapies into their work practice and that, in general, these healthcare formats are considered useful only for cases involving dependent people, people with mobility restrictions, caretakers of chronically ill people or adolescents [19], as well as people living in remote rural areas [5].

This article is part of the research area on therapies using mobile communication systems among vulnerable women’s groups in Spain. We applied psychosocial therapies with text messages to immigrant women in an intervention group and compared the results to the control group; this study was published in [20], where we demonstrated the effectiveness of text message therapy when combined with face-to-face therapy sessions. In this article, we analyze the effectiveness of text message therapies combined with face-to-face group therapies among female victims of gender violence. We followed the methodology for the design of a quasi-experimental investigation [21].

Based on the blended therapies, our research poses the following questions: Is it possible to integrate the smartphone into traditional face-to-face social service practice with female victims of gender-based violence? Are text messages combined with face-to-face group therapies effective in interventions with female victims of gender-based violence? What are the advantages and challenges of such integration? In this paper, we explore these questions, starting from a research project.

## 2. Materials and Methods

We focused on the design and evaluation of a blended therapy intervention, with the goal of improving the biopsychosocial health of a group of female victims of gender violence. We carried out an experience with a group of women living in the Autonomous Community of Galicia, Spain. Text message banks and face-to-face therapy group sessions were designed as intervention tools with a group of 39 female users of social services. These women owned personal mobile phones and were sufficiently capable of reading, understanding, and handling text messages.

The research, its method, objectives, and development were analyzed and approved by the Bioethics Committee of the University of Santiago de Compostela.

### 2.1. Procedure

We were handed information from the records of the Support Centers for the Care of Victims of Gender Violence in Galicia, and over a period of one month, we proceeded with the recruitment of the participants. All women were contacted and consented to attend a personal interview that was arranged in their municipality of residence.

A total of 42 women from 13 different municipalities were interviewed in person, and the characteristics and objectives of the research were explained to each of them. All, except one who refused, were receptive and interested in participating.

For each of the 41 women who signed the informed consent forms, we elaborated a psychosocial diagnosis with questions relating to concerns, health, medical treatment, social and family relationships, professional activities, economy, and physical activity. The information provided by each woman was complemented with the information obtained from her social history recorded in the public services. Based on all this information, we contacted each one by telephone to evaluate the messaging system to be used in each case. We found that the numbers were not operational in two cases. Therefore, after repeated contact attempts, the group was reduced to 39 cases (*n* = 39).

### 2.2. Text Messaging Apps

We designed two apps for scheduled messaging: HealthySMS for SMSs and ConHealth for internet-based messages. Both are managed from a web page that allows for the organization of information, management of technical issues, scheduled sending and receiving of messages and monitoring of personal health and moods.

Researchers from the School of Social Welfare at the University of California-Berkeley collaborated in the development of these apps. The University of California-Berkeley’s automated text messaging system for psychosocial therapies (https://www.moodtext.org, accessed on 28 December 2021) was previously used in a California hospital with small groups of vulnerable people in psychological therapeutic treatment [12,15] and with groups of long-term unemployed women in Santiago de Compostela [22].

The designed apps were managed through the internet: https://www.spain.healthysms.org (accessed on 28 December 2021) for the SMS system and https://www.conhealth.audacious-software.com (accessed on 28 December 2021) for the Android mobile application. The mobile app can be downloaded directly from Google Play to the phone, requiring permissions and a personal activation code that we provided privately to each participant.

In some cases, we used text messages to the telephone (*n* = 18), due to the use of old models without internet access, internet connection issues in their places of residence, or, in two cases, due to the use of the IOS-iPhone operating system. In the remaining cases (*n* = 21) the messages were sent via the internet. Most women, 34 cases (87, 2% of *n* = 39) had previously downloaded and installed apps from Google Play.

It is also important to note that we designed an alert system which instantly detected possible risks of suicidal or violent behavior, a system that can detect the presence of keywords in reply to messages. However, we did not detect alerts. We had an action protocol so that in a case of detection of an alert, we would urgently communicate it to the mental health services.

### 2.3. Face-to-Face Group Therapy

The women were free to participate in either the text message therapy intervention group (*n* = 18) or the text message and face-to-face therapy intervention group (*n* = 21). Face-to-face therapies took place in two scheduled sessions during January and February 2020 in small groups of 5–7 women and lasted an average of 2 h per session. The women who did not participate (*n* = 18) expressed interest, but had incompatible schedules due to work obligations, family obligations childcare, or geographical distance. These sessions were carried out by a single psychotherapist and were aimed at improving self-esteem, relaxation, communication skills, reflection, resilience, overcoming shyness, and managing emotions.

### 2.4. Telephone Contacts

Occasionally, we contacted with each woman throughout the intervention via telephone to build trust, address technical requirements, answer questions, and assess their emotional and personal health status.

We spent an average of 10 min per call. All women (*n* = 39) received seven telephone contacts: two contacts to install the app, four contacts to answer personal health and mood questionnaires, and one contact to request their health card personal information code. In eight cases (20.5% of *n* = 39) the app had to be reinstalled due to a change in the personal contact number or a change of phone to another model.

### 2.5. Text Messaging and Content

We carried out an individual test with each woman and respective mobile phone to ensure the correct reception of messages and subsequent replies. All of them were informed that they could reply at their own discretion and that the purpose of the messages was to monitor, advise and improve their moods and their physical and psychosocial health, stressing the need for them to read the messages and reflect on their content.

As to the financial costs, all women were told that receiving and replying to the messages was free of charge with the app and that SMS replies would be paid for. In nine cases the women were contacted via SMS.

For 120 days, from December 2019 to March 2020, we sent 2 text messages per day to the mobile phones of each woman at 09:00 AM and 21:00 PM. We had previously elaborated four message banks focused on thought, health, physical activity and social relationships, such as: “Some thoughts are unhealthy and contaminate our minds. Identify them and put them aside”, “How many pleasant activities have you done today?”, “Keeping a social life can make us feel better or worse”, “Try to identify the people who make you feel better”, and “What did you do today to take care of your health and wellbeing”. In total, each women received 240 text messages.

### 2.6. Measurements

To monitor and evaluate the effect of the intervention, we used two questionnaires and two indicators. On the one hand, we used a Personal Health Questionnaire (PHQ9) that allowed us to identify the mood and symptoms of depression at the beginning, in the middle, and at the end of the 120-day intervention period. The PHQ9 (Personal Health Questionnaire-9) is a widely validated instrument [23]. In addition, we used the validated instrument named Final Questionnaire on Text Messaging (FQTM) for each woman to evaluate her experience [12]; we asked questions about the positive, the negative, and the effectiveness of the intervention, their opinion on the number of messages received, changes in their daily lives, and their interest in continuing to receive messages. We also used as an indicator the number of replies recorded in the system for each woman.

To assess the individual compliance to the prescribed medication, we used the validated instrument named the SMAQ personal response questionnaire [24], and we carried out a statistical analysis of the data on prescribed medication available in the records of the public health service of the Autonomous Community of Galicia, for a period of 9 months, between October 2019 and June 2020.

### 2.7. Participants

In terms of social characteristics (see Table 1), these women (*n* = 39) shared a history of gender-based violence and were recognized as such by the public social services. Of these, 71.8% (*n* = 31) received social assistance and 64.1% (*n* = 25) were urban residents. Their average age was 42.5 years (SD = 10.6; range = 22–65) and they had an average of 1.7 children (SD = 1.3; range: 0–5). Most of them (84.6%, *n* = 33) were mothers with children in their care and, of these, 61.5% (*n* = 24) had minors. The average number of years of cohabitation as a couple was 11.2 per woman (SD = 10.0; range = 0.4–34) and of single cohabitation with couple separation was 2.3 years per woman (range = 0.6–14). All of them owned a mobile phone and the majority (71.8%, *n* = 28), were able to use the internet and manage social networks and apps. These women formed a heterogeneous group in terms of social characteristics: 20.5% (*n* = 8) were immigrant women from Colombia, Venezuela, Portugal, Cuba, Egypt, Uruguay, and Brazil and the majority (79.5%, *n* = 31) were Spanish. It is important to note that most had mental health problems and received psychological or psychiatric care (74.3%, *n* = 29). A total of 46.1% (*n* = 18) came from dysfunctional families, and 30.8% (*n* = 12) suffered from a chronic illness. The vast majority revealed psychosocial issues relating to uncertainty (94.8%, *n* = 37) and social relationship issues (92.3%, *n* = 36), as well as family related relationship issues (56.4%, *n* = 22).

To a lesser extent, there were cases of prostitution (12.8%, *n* = 5), drug dependence (15.4%, *n* = 6), and disability (10.2%, *n* = 4). As to their educational level, 35.9% (*n* = 14) completed primary school, 53.8% (*n* = 21) secondary school, 7.7% (*n* = 3) higher education, and only 2.6% (*n* = 1) had no education. Most were divorced or separated (48.7%, *n* = 19), with 43.6% (*n* = 17) single, and only 7.7% (*n* = 3) married.

We observed the vulnerability status of these women related to situations of precarious employment and low economic resources. The majority (87.1%, *n* = 34) were employed in low-skilled, sometimes informal jobs, such as caring for dependents, elderly or disabled people, house cleaning, agricultural work, and work in the hospitality industry or in commerce. Social characteristics were the same for both groups: the intervention group with text messages and group therapy (*n* = 21) and the intervention group with messages without group therapy (*n* = 18).

Attendance to group therapies involved travelling from the place of residence to the cities of Santiago de Compostela or Vigo. We observed that women with jobs and school-age children participated less, due to work and school schedule incompatibilities. In the group of women who received messages and group therapy, we detected a higher prevalence of cases under psychological/psychiatric treatment (85.7%), unemployed or without any current activity (52.4%), and with chronic illness or disability. They also participated more, with 38.1% and 14.3% compared to 22.2% and 5.5% for the group that received therapy with messages only (see Table 1).

In the initial interview, we developed a personal health questionnaire and did not detect any women with an optimal status (PHQ9 = 0); the range of PHQ9 scores was between 3 and 34 points.

### 2.8. Medication Adherence

In terms of adherence to the medication prescribed by the medical services (see Table 2), there was a balance between the number of women who did and did not take medication in the month preceding the intervention; 51.3% (*n* = 19) did not take medication and 48.7% (*n* = 19) answered that they had. Adherence to medication (*n* = 19) as prescribed by a doctor was low, as about half of the women, 48.7% (*n* = 8), reported having forgotten to take it at some point during the previous month. However, it should be noted that when asked how many times they had forgotten to take the medication in the previous week, 87.5% (*n* = 7) said once or twice, and only 1 reported more than 3 days.

We also detected a low adherence to medication in terms of schedule, prescribed frequency, and concern about forgetting to take the medication. As shown in Table 2, the majority (57.9%, *n* = 11), indicated that they did not take their medication at the prescribed time, and this mostly occurred at the weekends, when 87.5% (*n* = 7 out of *n* = 8) stated that they forgot to take their medication. The fact that they indicated feeling emotionally affected when they forget to take their medication (62.5%, *n* = 5 out of *n* = 8) is also noteworthy.

## 3. Analysis of the Results

During the 120-day period of psychotherapeutic intervention, all women (*n* = 39) replied to messages or phone calls; one woman only answered our phone calls and all others replied via text messages and phone calls (97.4%, *n* = 38). The case of the woman who did not reply to text messages involved an elderly person using an old phone.

Each woman (*n* = 39) received 240 personal messages on her mobile phone. We only sent more than the scheduled messages when asked specific questions about the operation of the system or when information was requested. A total of 11,597 messages were sent, of which 9120 (78.6%) were scheduled psychotherapeutic content and 2477 (21.4%) were replies to questions about the operation of the system or information requests.

In terms of replies to texts, we received 4913 over the course of the 120 days, representing an average of 129.3 written replies per woman (*n* = 38) (SD = 105.1; range = 2–365, IQR was 51). Women claimed that they did not reply sometimes because they were unsure of their answers, did not know what to say, were too busy with work or caring for children, or delayed the answer and forgot to reply. Most texts (88.0%) were replied within 15 min. In one case (2.6%, *n* = 39), the woman claimed that she was not always be able to reply due to problems with the prepaid phone when the available credit was exhausted.

### 3.1. Satisfaction towards Received Messages and Group Therapies

As to the questionnaire on satisfaction towards the received messages, 93.2% of the women (*n* = 36) replied (see Table 3). The majority indicated that the message therapy made them feel more connected to their social environment, with 80.6% (*n* = 29) agreeing or strongly agreeing with this statement. Moreover, most (91.6%, *n* = 33) agreed or strongly agreed with the statement that the messages had improved their mood and that they would like to continue receiving more messages (100.0%, *n* = 36), with a frequency of 2 to 3 messages per day (88.9%, *n* = 32). More significantly, 97.2% (*n* = 35) reported saving the messages to read them again, which reflects the value and usefulness attached to the messages by the subjects.

A total of 21 women (53.8% of *n* = 39) participated in the 2 face-to-face group therapy sessions. All of them (100%) were very satisfied with their participation, and the sessions helped to build trust and mutual support amongst the women. All the women reported feeling integrated and satisfied by sharing personal experiences and hearing first-person testimonies of other women who had experienced gender-based violence. They shared experiences and feelings about common problems, especially concerns about the slowness of legal procedures towards their complaints of gender violence, non-compliance with restraining orders issued against their aggressors, financial problems due to lack of income to access housing, unemployment or informal employment, and fears about leaving their children at the meeting points with their abusive fathers, out of obligation.

### 3.2. Replies to Messages

We proceeded with the evaluation of the quantifiable ratios of messages responded to by women. Over the 120-day intervention period, the reply rate to our text messages was 42.4% (SD = 34.7%, with a range of 0.6–119.3%). The Pearson correlation between age and the number of messages replied to is positive, so there is a tendency for older women to reply more than younger women (r = 0.36). It should be noted that all the participants had the necessary knowledge to use their mobile phones.

We found no significant correlations between the PHQ9 and the number of replies. The sample size limited the analysis of correlations between variables, paired difference tests, and other statistical comparisons.

### 3.3. FQTM: Final Questionnaire Text Messaging

In terms of qualitative responses to the Final Questionnaire on Text Messaging (FQTM) all women rated their personal experience (100%, *n* = 39).

The following questions were asked: “How did the messages help you?”, “What did you like the least?”, and “What would you improve?” The responses were conceptually analyzed using the grounded theory [25] open coding method, which, using an inductive process, allowed us to associate and condense the messages into four main thematic blocks:

(a) Thinking, with comments about mood improvement, relaxing, thinking things through, seeing things from a different perspective, dealing with problems, being more confident, improving motivation and reasoning, and having more concentration or the ability to reflect.
(b) Organization, with references to planning day to day lives, setting goals, changing habits and attitudes, increasing motivation, becoming more aware and consistent, or doing things by themselves.
(c) Monitoring, feeling counselled, supported, and improving family and social relations.
(d) Healthy habits, with answers about improving their diet, overcoming depression, reducing anxiety, remembering to take medication, exercising more, or taking better care of themselves.

All women (100%, *n* = 39) stated that they liked receiving the messages and would like to continue receiving them. In terms of what they would improve and what they disliked the most, two cases specified that they were sometimes repetitive (5.1%, *n* = 39), three said they would like to receive messages to help them rethink the past and overcome guilt (7.7%, *n* = 29), three stated they would prefer more personalized and face-to-face care (5.1%, *n* = 39), and two mentioned that they would like to talk more in person (5.1%, *n* = 39). No problems or inconveniences were reported in terms of timing or message content.

### 3.4. PHQ-9: Personal Health Questionnaire

Depressive symptoms and mood states were assessed using the Personal Health Questionnaire-9 (PHQ-9) at the beginning, middle, and end of the intervention. Scores of 5 denote mild depression, scores above 5 and up to 15 denote moderate depression, and scores above 15 denote severe depression [23].

**Figure 1:**
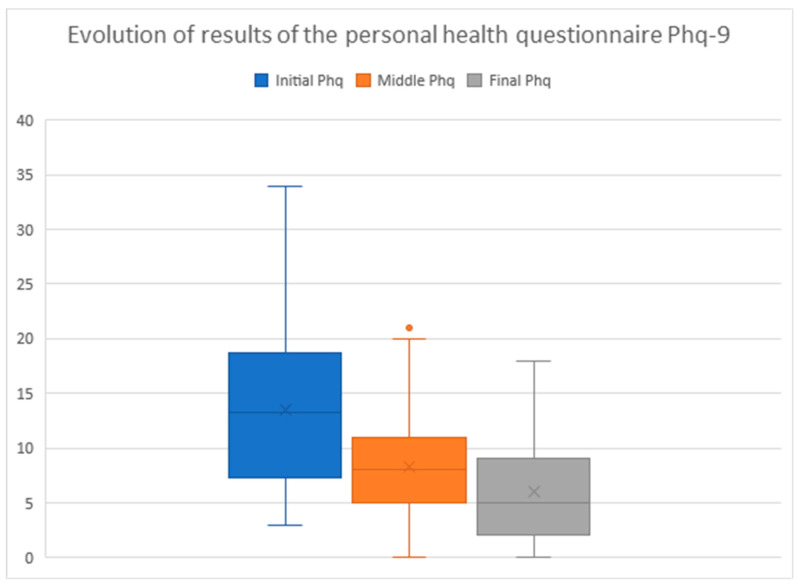
Data representation of the initial, middle, and final PHQ-9 (*n* = 39).

As a whole (*n* = 39), the average group results were highly positive and evolved steadily (see Figure 1), starting from an initial PHQ-9 of 13.5 (SD = 7.4, range 3–34), improving to 8.3 in the intermediate phase (60 days) (SD = 5.4, range 0–21), and improving again to 6.0 (SD = 5.2, range 0–18) at the end of the message period (120 days). There was a significant difference between the initial and final PHQ-9 value (*p* = 8.59956 × 10^−10^). In 38 of the cases (97.4%) the PHQ9 decreased between the initial and final values, while in 1 case it remained unchanged (2.6%).

In the subgroups of women who used the ConHealth app, via the internet (*n* = 29), and those who used SMS (*n* = 10), the changes between the initial, intermediate, and final PHQ-9 values were similar, and there were no significant differences. In the internet group, the value changed from an initial PHQ-9 of 13.0, to an intermediate value of 7.9, and a final value of 5.6 (SD = 6.4; 4.9; 5.5. Ratio 3–25; 0–20; 0–18, *p* = 1.86626 × 10^−7^). In the SMS group the value changed from an initial PHQ-9 of 16.1 to an intermediate value of 10.2 and a final value of 7.7 (SD = 9.1; 6.4; 3.8. Ratio 3–34; 1–21; 0–13, *p* = 0.000). A constant and sustained improvement was observed in both groups, regardless of the technology used.

As to women who received messages and group therapy (*n* = 21) and those who received message therapy only (*n* = 18), the changes in PHQ-9 value were positive and significant in both cases. In the message and therapy group, the PHQ-9 changed from 13.2 to 8.1, and, finally, to 6.1 (SD = 6.1, 3.6, 5.2. Ratio 3–22; 1–14; 0–18, *p* = 6.50294 × 10^−6^). In the message-only group, the PHQ-9 changed from 13.7 to 8.5 and, finally, to 5.8 (SD = 8.4; 6.9; 5.4. Ratio 3–34; 0–21; 0–16, *p* = 5.77615 × 10^−5^). However, comparatively the data were significantly better in the message group with group therapy.

### 3.5. Anxiolytics, Antidepressants, and Hypnotics

Regarding changes in prescribed medications, we sorted and classified the data on the number of packages sold in pharmacies with official prescriptions. A total of 23 women, representing 58.9% of all participants (*n* = 39), shared with us their health card personal identification code (PIC). The women who refused to provide their PIC mentioned privacy concerns. We found a direct relationship between participation in the face-to-face group therapies and personal willingness to provide the PIC code; 21 women out of the 23 who allowed access to their personal medication data attended the group therapy sessions organized during the intervention period (91.3%). It was not possible to make comparisons between the message group with face-to-face therapy and the message-only group.

The public health service provided us with data on medication billed between October 2019 and June 2020, covering a period of 9 months. We defined 3 periods of 3 months, a pre-intervention period from October to December 2019, an intervention period from January to March 2020, and a post-intervention period from April to June 2020.

We identified 12 cases of women prescribed with anxiolytic medications (52.2% of *n* = 23), 7 cases prescribed with antidepressants (30.4% of *n* = 23), and 4 cases prescribed with hypnotics (17.4% of *n* = 23). The results show a decrease in all three types of medication between the pre-intervention and post-intervention periods (see Table 4): anxiolytics went from an average of 1.19 to 1.11 packs per woman, antidepressants went from 1.19 to 1.10, and hypnotics went from 1.00 to 0.75. The decrease in medication was coincident with the positive changes observed in the Personal Health Questionnaire, PHQ-9 (see Table 4), for the same period. In the anxiolytic group of women, the PHQ-9 changed from an initial, or pre-intervention, value of 14.25 to a final, or post-intervention, value of 7.75; in the antidepressant group, the PHQ-9 went from 16.00 to 11.14; and in the hypnotic group the value decreased from 14.50 to 8.00.

The *t*-test shows that positive changes in the PHQ-9 were statistically significant in all three medication groups between the pre-intervention and post-intervention periods: anxiolytics *p* = 0.001, antidepressants *p* = 0.017, and hypnotics *p* = 0.004. No statistically significant changes were observed in the prescribed medication: anxiolytics *p* = 0.680, antidepressants *p* = 0.7476, and hypnotics *p* = 0.081; further investigations with a larger number of cases are necessary.

We controlled for possible biases in medication packaging. We identified changes in the number of capsules and mg of active substance per pack prescribed for each woman. The general tendency was to keep the medication unchanged. There were only two changes in the anxiolytic group, one switching from pregabalin 75 mg to 25 mg per capsule and one switching from lorazepam 1 mg 50 capsules to 1 mg 25 capsules.

### 3.6. The Mood

**Figure 2:**
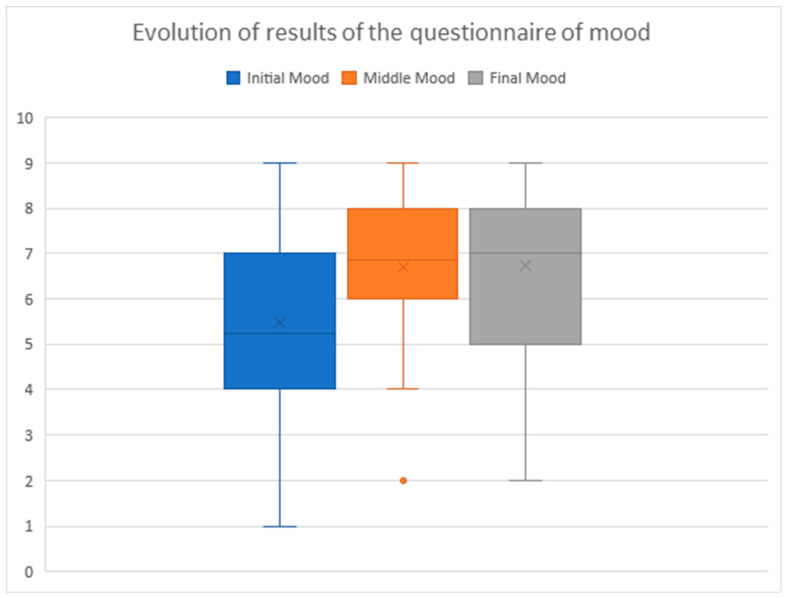
Data representation of the initial, middle, and final mood (*n* = 39).

The mood of the entire group (*n* = 39) evolved positively (see Figure 2). It was measured on a Likert scale from 1 to 9, using the specific questionnaire PHQ-9 administered by telephone at the beginning, middle, and end of the 120-day intervention, where 1 is the lowest value and 9 the highest.

The average was 5.4 in the beginning, 6.7 in the middle, and 6.8 by the end, whereby the improvement in mood was sustained and the change was statistically significant (SD = 2.2; 1.6; 2.1. Ratio 1–9; 1–9; 2–9, *p* = 0.007) (see Figure 2).

We did not find differentiated and significant data in the groups according to the technology used—internet/SMS—or whether they had participated or not in the group therapy sessions. It should be noted that 64.1% of the women showed an improved mood by the end, when compared with their initial one (*n* = 25), in 5 cases they maintained their initial values (12.8%), and in 9 cases their initial values decreased (23.1%).

## 4. Discussion

The obtained results show that text messages are effective in reducing anxiety and depression in female victims of gender violence, increase adherence to medication, and are positively valued by these women. Therefore, the obtained results provide validation for cases of gender violence and reinforce the conclusions of previous research carried out with immigrants, the long-term unemployed, people with addictions, alcoholism, etc., [9,10,11,12,13,14,15,16,17,20].

Face-to-face group therapy complements the psychosocial intervention with text messages, reinforces support and mutual knowledge among the participants, and promotes communication, as well as a psychotherapeutic bond. However, group therapy sessions should be organized in locations that are accessible and close to the place of residence of the participants to avoid long journeys and favor professional and family conciliation. We only carried out two group therapy sessions and we cannot draw conclusions about their influence on the obtained results. However, we must consider that the women who have provided us with their medication data have mostly been the participants in face-to-face group therapies. Therefore, we believe that the therapeutic alliance could have been greater.

In terms of possible limitations, we found that there were no financial issues attached to costs; messages were received and replied to, free of charge, through the app that was designed. All women had an internet connection on their phones except for one case of an elderly woman with an old mobile phone. For low-income people, this may pose a constraint that forces them to look for free Wi-Fi access points, with the associated costs in terms of time and/or mobility. Digital inequality, in terms of access to resources and services, is also limitation.

We sent text messages with advice, guidance, and key questions to stimulate reflection based on the data obtained from face-to-face contacts and telephone contacts with each of the women. It was possible to group and link recipients and messages by the degree of depression and mood of the recipient measured with the PHQ-9 to guide positive changes in behavior. Our study followed a quasi-experimental methodology and we obtained positive results comparable to those previously achieved with immigrant women [26], but the group of female victims of gender-based violence was not homogeneous in terms of age, country of origin, education, employment, or marital status.

We must continue with further research to design specialized text message banks based on cultural, social, economic, and demographic diversity. It is necessary to extend the reception periods of messages over 90 days and offer a greater number of face-to-face group therapy sessions to assess the continuity and permanence of the positive changes achieved in moods and symptoms of depression. In new investigations of blended therapy, it is necessary to expand the number of participating female victims of gender violence and include a control group that does not receive messages. Questions remain about the effectiveness of text message therapy, group therapy, and social variables, such as marital status, urban-rural origin, nationality, etc. The small sample size of study participants did not allow this comparison.

We spent an average of 30 min in face-to-face interviews and 35 min in phone calls (7) with each of the participating women (*n* = 39), representing a relatively low cost for a public social inclusion service. Mobile phone text messages with scheduled delivery systems can promote more frequent care and a more personal interaction time for the social services professionals with female victims of gender-based violence.

As to future research, questions arise about psychosocial interventions through blended therapies with vulnerable social groups in other countries and cultural communities, which calls for further research formulated from the perspective of the social services.

We have no doubt that specialized software for blended therapies will have to be developed by social services professionals in an interdisciplinary collaboration with computer engineers, healthcare professionals, social workers, psychologists, and other social scientists, as stated by García, Ferrás, and Ginzo [26].

We also agree with Steiner [8], who believes that digitalization should be a fundamental part of the training and qualification of future social services professionals, not only in terms of use but also in terms of ethical and data protection issues, which should be subject to more studies [8,27].

## 5. Conclusions

With the blended therapies and mobile phones, social services professionals can incorporate technology into daily practices. Text messages open possibilities for personalized care, counselling, support, and guidance for vulnerable population groups. The use of text messages by social services helps provide personalized services on a daily, more accessible, and universal basis. They also allow for the provision of specialized services to vulnerable people in both rural and urban areas, avoiding travel and saving time and money for users and the administration. In the context of the COVID-19 pandemic, blended therapy and mobile phones can facilitate remote care, avoiding travel and the daily physical presence in health and social centers.

The results demonstrate the practical feasibility of the blended therapies. Scheduled text messages and face-to-face group therapy improve education, mood, and medication adherence and promote behavioral changes and the biopsychosocial health of female victims of gender-based violence.

We can, therefore, say that it is possible to integrate smartphones into the face-to-face social services practice with female victims of gender-based violence; we showed that text messages combined with face-to-face group therapy are effective in this type of intervention. The advantages include the possibility for more frequent and personalized psychosocial care and avoiding unnecessary travel. However, there are also challenges in the development of social-services-specific software, the digital training of professionals, interdisciplinary collaboration, research into ethical and data protection issues, and practical research into blended therapies with other vulnerable groups and individuals.

## Figures and Tables

**Table 1:** Characteristics of the participating women (*n* = 41).

Characteristics	Intervention with Messages and Group Therapy (*n* = 21)	Intervention with Messages without Group Therapy (*n* = 18)	Total Both Groups (*n* = 39)
Average age (years)	43.0	41.8	42.5
Average number of children	1.8	1.5	1.7
With minor children	57.1% (*n* = 12)	66.6% (*n* = 12)	61.5% (*n* = 24)
Immigrants	19.0% (*n* = 4)	22.2% (*n* = 4)	20.5% (*n* = 8)
Comes from an unstructured family	42.8% (*n* = 9)	50.0% (*n* = 9)	46.1% (*n* = 18)
Gender violence ^1^	100.0% (*n* = 21)	100.0% (*n* = 18)	100.0% (*n* = 39)
Years of separation from the couple	2.6	2.2	2.3
Years of living together as a couple	12.3	10.0	11.2
With chronic disease	38.1% (*n* = 8)	22.2% (*n* = 4)	30.8% (*n* = 12)
With handicap	14.3% (*n* = 3)	5.5% (*n* = 1)	10.2% (*n* = 4)
With uncertainty problems	90.2 (*n* = 20)	94.4% (*n* = 17)	94.8% (*n* = 37)
Drug addiction (cases)	14.3% (*n* = 3)	16.7% (*n* = 3)	15.4% (*n* = 6)
Prostitution (cases)	9.5% (*n* = 2)	16.7% (*n* = 3)	12.8% (*n*= 5)
With social relationship problems	90.5% (*n* = 19)	94.4% (*n* = 17)	92.3% (*n* = 36)
With family relationship problems	52.4% (*n* = 11)	61.1% (*n* = 11)	56.4% (*n* = 22)
Urban residence ^2^	61.9% (*n* = 13)	66.6% (*n* = 12)	64.1% (*n* = 25)
Unemployed or without current Activity	52.4% (*n* = 11)	33.3% (*n* = 6)	43.6% (*n* = 17)
Married	9.5% (*n* = 2)	5.5% (*n* = 1)	7.7% (*n* = 3)
Separated or divorced	47.6% (*n* = 10)	50.0% (*n* = 9)	48.7% (*n* = 19)
Single	42.8% (*n* = 9)	44.4% (*n* = 8)	43.6% (*n* = 17)
Psychological/Psychiatric Treatment	85.7% (*n* = 18)	61.1% (*n* = 11)	74.3% (*n* = 29)
Primary studies	23.8% (*n* = 5)	50.0% (*n* = 9)	35.9% (*n* = 14)
Secondary studies	66.7% (*n* = 14)	38.9% (*n* = 7)	53.8% (*n* = 21)
Higher education	9.5% (*n* = 2)	5.5% (*n* = 1)	7.7% (*n* = 3)
No studies	0.0% (*n* = 0)	5.5% (*n* = 1)	2.6% (*n* = 1)
Good smartphone handling	80.9% (*n* = 17)	61.1% (*n* = 11)	71.8% (*n* = 28)
Receives social assistance ^3^	85.7% (*n* = 18)	72.2% (*n* = 13)	79.5% (*n* = 31)

^1^ Gender violence refers to some moment in life. In all cases, they are women who have received assistance from social services as victims of gender violence. ^2^ An urban residence is in municipalities with more than 100 inhabitants/km^2^. ^3^ It refers to the fact that they received some type of economic benefit from the social services of the public administration.

**Table 2:** Data on adherence to medication in the last month before the psychosocial intervention.

(*n* = 39)	Yes	No
Do you take medication?	48.7% (*n* = 19)	51.3% (*n* = 20)
You ever forgot to take your medication?	42.1% (*n* = 8)	57.9% (*n* = 11)
Do you take your medication at the indicated time?	42.1% (*n* = 8)	57.9% (*n* = 11)
Do you feel bad when you don’t take your medication?	62.5% (*n* = 5)	37.5% (*n* = 3)
Do you forget to take medication on the weekend?	87.5% (*n* = 7)	12.5% (*n* = 1)
How many days in the last week did you forget to take your medication? (*n* = 19)		
None	57.9% (*n* = 11)	
1 to 2 days	36.8% (*n* = 7)	
3 to 5 days	5.3% (*n* = 1)	

**Table 3:** Final questionnaire on text messages (FQTM).

Questions Answered (*n* = 36)	Disagreement	Neutral	Agreement	Strongly Agree
Did the messages make you feel more connected to your social environment?	0.0% (*n* = 0)	19.4% (*n* = 7)	13.9% (*n* = 5)	66.7% (*n* = 24)
Did the messages boost your mood?	0.0% (*n* = 0)	8.3% (*n* = 3)	19.4% (*n* = 7)	72.2% (*n* = 26)
	**Yes**	**No**		
Would you like to continue receiving the messages?	100% (*n* = 36)	0.0% (*n* = 0)		
Do you save received messages?	97.2% (*n* = 35)	2.8% (*n* = 1)		
	**1**	**2 or 3**	**4 or more**	
What number of messages would you like to receive each day?	11.1% (*n* = 4)	88.9% (*n* = 32)	0.0% (*n* = 0)	

**Table 4:** Medicines with an official prescription from the health service dispensed between October 2019 and June 2020.

Anxiolytics (*n* = 12)				
	Pre-Intervention	Intervention	Post-Intervention	*T*-Test
No. containers	43	40	40	*p* = 0.680
Average containers per woman	1.19	1.11	1.11	*p* = 0.680
PHQ-9	14.25	8.83	7.75	*p* = 0.001
**Antidepressants (*n* = 7)**				
	**Pre-Intervention**	**Intervention**	**Post-Intervention**	***T*** **-Test**
No. containers	25	20	23	*p* = 0.747
Average containers per woman	1.19	1.00	1.10	*p* = 0.747
PHQ-9	16.00	9.86	11.14	*p* = 0.017
**Hypnotics (*n* = 4)**				
	**Pre-Intervention**	**Intervention**	**Post-Intervention**	***T*** **-Test**
No. containers	12	11	9	*p* = 0.081
Average containers per woman	1.00	0.92	0.75	*p* = 0.081
PHQ-9	14.50	7.50	8.00	*p* = 0.004

Prescription drugs: pregabalin, diazepam, alprazolam, lorazepam, and bromazepam. Prescription packages varied in several capsules and mg/capsule in two cases, one with pregabalin and the other with lorazepam, which reduced the mg/capsule and number of capsules per package between the pre-intervention and post-intervention periods. Prescription drugs: venlafaxine, amitriptyline, duloxetine, quetiapine, mirtazapine, vortioxetine, bupropion, methylphenidate, fluoxetine, and paroxetine. There were no variations in the packages in terms of mg/capsule and number of capsules. Prescription drugs: lormetazepam. There were no variations in the packages in terms of mg/capsule and number of capsules. Pre-intervention: October, November, and December 2019. Intervention: January, February, and March 2020. Post-intervention: April, May, and June 2020.

## Data Availability

All the data presented in this study are available upon request from the corresponding author.
